# Stone fragment in the anterior chamber angle: a 40-year-old ocular emergency

**DOI:** 10.5935/0004-2749.2023-0030

**Published:** 2023

**Authors:** Kemal Örnek, Özkan Kocamış

**Affiliations:** 1 Department of Ophthalmology, Kırşehir Ahi Evran University School of Medicine, Kırşehir, Turkey

Dear Editor,

An intraocular foreign body (IOFB) is any material that penetrates the ocular tissue. This foreign material may be retained within the eye or projected out of the eye, as in high-velocity injuries. The presence of an IOFB could increase the risk of endophthalmitis and other ocular morbidities such as corneal scarring, cataract, retinal detachment, etc.^(1)^. The anterior chamber is the most common location for anterior segment IOFBs^(2)^.

In this report, we present an unusual case of an anterior segment non-metallic IOFB, which was incidentally discovered in the anterior chamber drainage angle of the right eye of a housewife 40 years after the inciting injury.

A 68-year-old woman was referred to the ophthalmology department with complaints of gradually decreasing visual acuity in her right eye for the last 12 months. There had been a history of ocular trauma approximately 40 years before the visit. She had not felt any discomfort; therefore, she had not consulted any physician.

**Figure 1:**
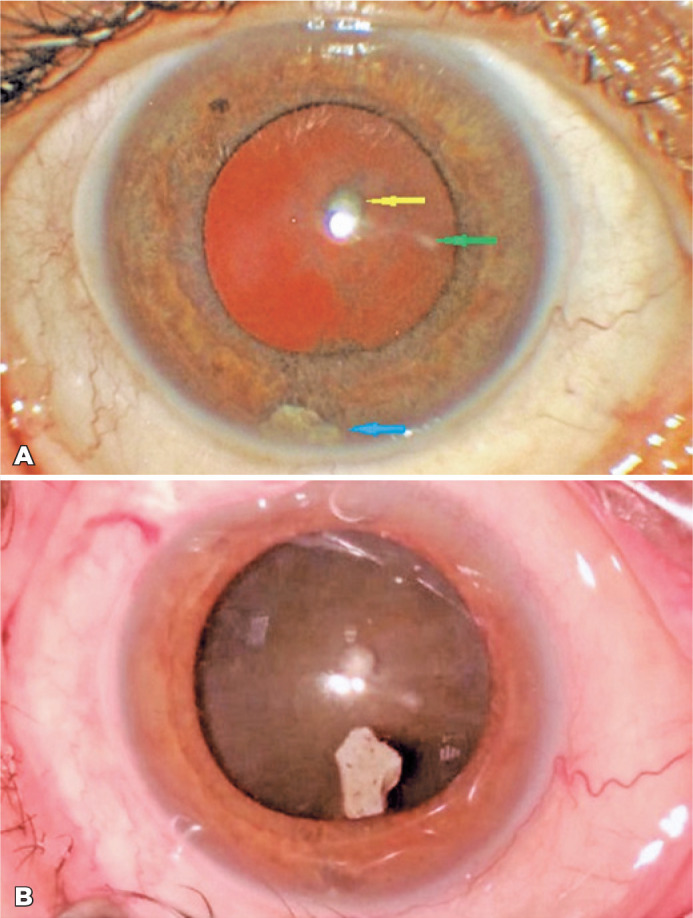
(A) Stone fragment in the lower iridocorneal angle (blue arrow), linear corneal scar (green arrow), and whitish spot on the anterior lens capsule (yellow arrow). (B) Stone fragment in the anterior chamber before removal.

On her examination, the best-corrected visual acuity was 2/10 in the right eye and 7/10 in the left eye. The intraocular pressure was determined to be 16 mmHg and 17 mmHg in the right and left eyes, respectively. There was no relative afferent pupillary defect, and the pupils were reactive bilaterally. On slit-lamp examination, there was an old paracentral linear scar with sharp borders measuring 3 mm on the cornea. The anterior chamber was quiet. At the 6 o’clock position of the iris, a stone fragment without any membranous capsule was found to be fixed to the anterior chamber drainage angle (Figure 1A). There were no posterior synechiae at the pupillary border. The lens examination revealed a whitish spot on the anterior capsule corresponding to the corneal scar and nuclear cataract without zonular dehiscence. The fundus examination was normal.

Before the cataract surgery, the foreign body was extracted through the main corneal incision via tying forceps (Figure 1B). The iris was found to be perforated at the 6 o’clock position. There were no intraand post--operative complications. The recent visual loss in the patient improved after surgery.

Intraocular foreign bodies are one of the most common causes of open globe injuries^(3)^. Stone is among the commonest materials causing ocular trauma^(2,4)^. Ocular injuries due to IOFBs are variable in presentation, outcome, and prognosis, depending upon several factors. IOFBs can cause direct damage to the ocular tissues but can also bounce in the eye, causing further damage, as in this case. Fortunately, after bouncing off the lens surface, the stone fragment dropped into the lower anterior chamber angle and remained there for 40 years. Further damage depends on the composition of IOFB. For instance, metallic IOFBs can cause siderosis or chalcosis. Inert substances such as stone, glass, or plastic are better tolerated than metals^(5)^. In addition, the mechanism of entry and the object’s size are also factors that determine the extent of the injury. IOFBs entering the sclera usually cause more damage than those entering the cornea^(6)^. As in this case, high-velocity and small IOFBs cause a minor linear laceration that is less damaging than blunt trauma.

A detailed history is essential in ocular trauma to discover the type or material of the IOFB and the mechanism of injury. Some patients may be asymptomatic or often report a faint sensation of something hitting the eye without any visible ocular changes, so that the event may be neglected for years.

To conclude, this report reveals a rare possibility of intact vision and anatomy in the presence of an IOFB. Without infection, the eye may remain quiet for years with a stone fragment in the anterior chamber.
